# Radiotherapy is associated with significant improvement in local and regional control in Merkel cell carcinoma

**DOI:** 10.1186/1748-717X-7-171

**Published:** 2012-10-17

**Authors:** Susan H Kang, Lauren E Haydu, Robin Yeong Hong Goh, Gerald B Fogarty

**Affiliations:** 1 Faculty of Medicine, University of New South Wales, Botany Street, Sydney, NSW, 2052, Australia; 2 Research and Biostatistics, Melanoma Institute Australia, 40 Rocklands Rd, Crows Nest, NSW, 2065, Australia; 3 Genesis Cancer Care, St Vincent’s Clinic, 438 Victoria Street, Darlinghurst, NSW, 2010, Australia; 4 Radiation Oncology, Mater Hospital, 25 Rocklands Rd, Crows Nest, NSW, 2065, Australia

**Keywords:** Merkel cell carcinoma, Radiotherapy, Immunosuppression

## Abstract

**Introduction:**

Merkel cell carcinoma (MCC) is a rare tumour of skin. This study is a retrospective audit of patients with MCC from St Vincent’s and Mater Hospital, Sydney, Australia. The aim of this study was to investigate the influence of radiotherapy (RT) on the local and regional control of MCC lesions and survival of patients with MCC.

**Method:**

The data bases in anatomical pathology, RT and surgery. We searched for patients having a diagnosis of MCC between 1996 and 2007. Patient, tumour and treatment characteristics were collected and analysed. Univariate survival analysis of categorical variables was conducted with the Kaplan-Meier method together with the Log-Rank test for statistical significance. Continuous variables were assessed using the Cox regression method. Multivariate analysis was performed for significant univariate results.

**Results:**

Sixty seven patients were found. Sixty two who were stage I-III and were treated with radical intent were analysed. 68% were male. The median age was 74 years. Forty-two cases (68%) were stage I or II, and 20 cases (32%) were stage III. For the subset of 42 stage I and II patients, those that had RT to their primary site had a 2-year local recurrence free survival of 89% compared with 36% for patients not receiving RT (p<0.001). The cumulative 2-year regional recurrence free survival for patients having adjuvant regional RT was 84% compared with 43% for patients not receiving this treatment (p<0.001). Immune status at initial surgery was a significant predictor for OS and MCCSS. In a multivariate analysis combining macroscopic size (mm) and immune status at initial surgery, only immune status remained a significant predictor of overall survival (HR=2.096, 95% CI: 1.002-4.385, p=0.049).

**Conclusions:**

RT is associated with significant improvement in local and regional control in Merkel cell carcinoma. Immunosuppression is an important factor in overall survival.

## Introduction

Merkel Cell Carcinoma (MCC) is an aggressive cutaneous neoplasm with malignant neuroendocrine differentiation [1]. It was first reported by Toker in 1972. It is a rare tumour that predominantly afflicts the elderly white population with a male predominance [2-9]. MCC has early local invasion, nodal involvement, distant metastases and a high rate of recurrence [10-13]. There is controversy internationally in the relative roles of surgery and RT in treatment [14]. There have been no completed randomised controlled trials reported in this disease as yet. Treatment regimes are variable. As MCC has been shown to have high radiosensitivity, adjuvant RT (adj RT) is supported by a number of studies [7,15-17]. Surgery followed by adj RT to the primary site as well as the lymphatic basin is recommended by some previous studies [8,18-22]. Few studies examined the effect of immune status on of MCC [1,2,5,6,23]. This study is a single institutional retrospective study of consecutive patients with MCC at St Vincent’s and Mater hospitals, both tertiary referral hospitals in Sydney. The aim of this study was to investigate the influence of RT on the local and regional control of MCC lesions and survival of patients with MCC.

## Methods and materials

Research was carried out in compliance with the Helsinki Declaration and this project was approved by the St Vincent’s Hospital Human Research Ethics Committee. Patients with a histologically proven diagnosis of MCC who presented between January 1996 and June 2007 to the St. Vincent’s and Mater Hospitals, Sydney, Australia were identified by the International Code of Diseases index from medical records database in anatomical pathology, RT and surgical departments.

Patient tumour and treatment characteristics were collected and analysed. Patient factors include age, sex and any history of immunosuppression. Immunosuppressed patients were either six transplant patients [heart (3), lung (2) and kidney (1)] or eight patients that had been on long term steroids. They included seven patients with connective tissue diseases [Ulcerative colitis, Pyoderma gangredorsum, Scleroderma, Myasthenia gravis, Psoriatic arthritis, Rheumatoid arthritis and multiple myeloma] and one patient with long term kidney cancer. Some of these patients also had diabetes mellitus. There were no patients who had diabetes alone without either being a transplant patient or had not been on long term steroids. Tumour factors recorded included tumour site, size, and involvement of regional lymph nodes, distant metastatic disease and overall staging. Treatment characteristics recorded included whether wide local excision (WLE) is used or not, regional dissection, RT to the primary and regional sites and the use of chemotherapy both in the adjuvant and definitive settings.

### Staging

Patients were staged by a four-tiered staging system developed by Memorial Sloan-Kettering Cancer Centre (MSKCC) [8]. The system is the most commonly used staging system consistent with the American Joint Committee on Cancer. For staging, most patients underwent computed tomography (CT) of the chest, upper abdomen, and regional nodes.

### Radiotherapy technique

Our departmental policy of RT of MCC is detailed below.

#### RT treatment of the primary site

The primary site is treated with 46-50Gy in 2 Gy fractions to an area of skin that includes the lesion or scar with a five cm margin. Compromise may need to be made for nearby dose limiting organs e.g. eyes. An electron or superficial/orthovoltage technique is often used.

#### RT treatment of the regional lymph nodes

The target for regional treatment is the draining lymph nodes and is given with same dose using megavoltage techniques. Gross disease may be boosted to a higher dose e.g. to 60 Gy. Bolus is used to cover any scars or drain sites to achieve full dose on operated skin that is thought to be at risk. The field treating the primary is junctioned to the regional field if field edges are within 5cm and there is no danger of unnecessary toxicity. Axillary fields are treated as per Fogarty et al. [24].

### Statistical analysis

IBM SPSS Statistic v 19.0 was used to conduct all statistical analyses. Univariate survival analysis of categorical variables was conducted with the Kaplan-Meier method together with the Log-Rank test for statistical significance. All patient, tumour and treatment factors were tested for association with each survival outcome in univariate analysis and those found to be significant were then entered into a multivariate model. Continuous variables were assessed using the Cox regression method. A p-value less than 0.05 was considered statistically significant.

Local recurrence free survival was defined as the time from initial diagnosis to the first local recurrence or date of last follow-up. Regional recurrence free survival was defined as the time between initial diagnosis and first regional recurrence. The disease-free interval was defined as the months between the date of initial diagnosis with MCC and the patient’s first recurrence of MCC or date of last follow-up. MCC-specific survival (MCCSS) was defined as the interval between initial diagnosis and the date of last follow-up; death from MCC was considered an event, and all other cases were censored. Overall survival (OS) was assessed as the interval between initial diagnosis and date of last follow-up; death from MCC or other causes was considered an event.

## Results

### Patient characteristics

Sixty-seven patients with MCC were identified. Five patients presented stage IV disease. These five patients were treated with palliative intent and were excluded from the analysis. The reported analysis is therefore a study of radical treatment of 62 patients with stage I-III disease.

Patient and clinicopathological characteristics are listed in Table 1. The overall analysed cohort of 62 patients was 68% male, 32% female, and had a median age at diagnosis of 74 years. Forty-two cases (68%) were stage I or II. Twenty cases (32%) were initially diagnosed with involved lymph nodes (stage III).

**Table 1:** Summary characteristics for the patient cohort

**Factor**	**Value**	**N**
Patient sex	Male	42
	Female	20
Age at Initial Diagnosis	(years)	Median=74 (Range:47-88)
Immunocompromised at initial diagnosis	Yes	14
	No	48
Types of immunosuppression	Long-term steroids	8
	Transplantation	6
Primary site of MCC	Head & Neck	32
	Upper limb	8
	Lower limb	9
	Trunk	3
	Buttocks	1
	Not known	9
Primary macroscopic size	(mm)	n=53; Median=15 (range 5-60mm)
Stage	I	38
	II	4
	III	20
Lymphadenectomy	Yes	17
	No	45
Final surgical treatment for primary	Incisional biopsy	3
	Excisional biopsy	21
	WLE	29
	Not applicable	9
Adjuvant RT to the Primary site	Yes	43
	No	10
	Not applicable	9
RT to the Regional Node site	Yes	43
	No	19
Local recurrence	Yes	9
	No	53*
Regional recurrence	Yes	16
	No	46
Distant recurrence	Yes	16
	No	41
	Not known	5
Status at Last Follow-up	Alive	22
	Died of Disease	20
	Died of other/Unknown	20

*Out of 53 with no local recurrence, 9 unknown primaries are included.

Forty-three patients (69%) received adjuvant RT to their primary site. The other 19 patients did not receive primary site RT. Of these 19 patients, 9 presented with nodal disease only, with unknown primary site. The remaining 10 were stage I (n=9) and stage II (n=1). Thirty two stage I and II cases received adjuvant RT to their primary site which was completed in a median of 79.5 (range 49–338) days after the diagnosis of the primary MCC. Out of these 32 patients, 25 additionally received prophylactic RT to their regional nodes completed in a median of 78 (range 54–338) days from the date of primary diagnosis. There was no significant or unexpected RT treatment toxicity reported for this cohort. Twenty patients died of disease.

Table 2 further describes the treatment patterns for the primary MCC. Three patients received incisional biopsy with positive margin status and all received RT.

**Table 2:** Treatment Patterns for Primary MCC

**RT Adjuvant to Primary Site**	**Incisional biopsy**	**Excisional biopsy**	**WLE**	**Unknown Primary Site**	**Total**
Yes	3	16	24	-	43
No	-	5	5	-	10
NA	-	-	-	9	9

### Staging

Of the 20 stage III patients, 19 were clinically positive; one clinically negative patient had positive 1^st^ echelon nodes on histology after sampling. Fifteen of these 20 had lymphadenectomy and adj RT, 3 patients received RT only to the nodes, and 2 patients had lymphadenectomy only. There were 7 patients who received chemotherapy, 5 of them were stage III patients. All these patients received chemotherapy concurrently. Only 1 of 67 had Positron Emission Tomography (PET) scan. Sentinel Lymph Node Biopsy (SLNBx) was done in only 1 patient.

### Recurrences

Nine patients (14%) experienced a local recurrence of their MCC. Sixteen (26%) developed a regional recurrence; all loco-regional recurrences were observed in stage I or II patients. No stage III patients had regional relapses observed with a median follow-up of 44 months (range 7–115 months). Distant recurrence status was known for 57 patients and, of these, 16 (28%) recurred in a distant site (11 stage I/II; 5 stage III).

### Impact of radiotherapy on survival

#### Local recurrence free survival

**Figure 1:**
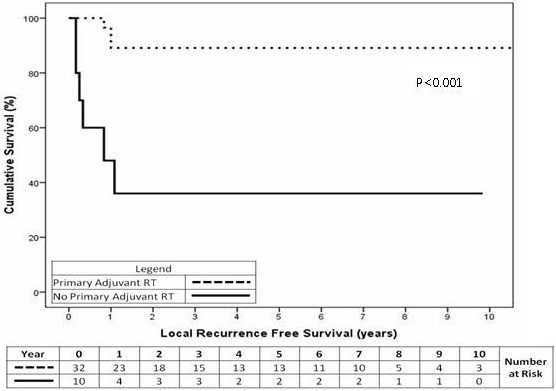
Local recurrence free survival in stage I and II patients having adjuvant RT to their primary site (p<0.001).

For the subset of 42 stage I and II patients, those that had RT to their primary site (n=32) had a 2-year local recurrence free survival of 89% compared with 36% for patients (n=10) not receiving RT (Figure 1, p<0.001).

#### Regional recurrence free survival

**Figure 2:**
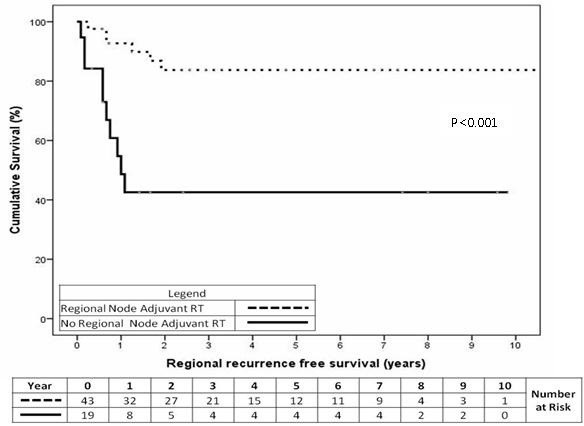
Regional recurrence free survival for the overall cohort for patients having adjuvant or prophylactic RT to their regional nodes (p<0.001).

The cumulative 2-year regional recurrence free survival for patients (n=43) having adj regional RT was 84% compared with 43% for patients (n=19) not receiving this treatment (Figure 2, p<0.001).

#### Disease-free survival for RT to primary site

**Figure 3:**
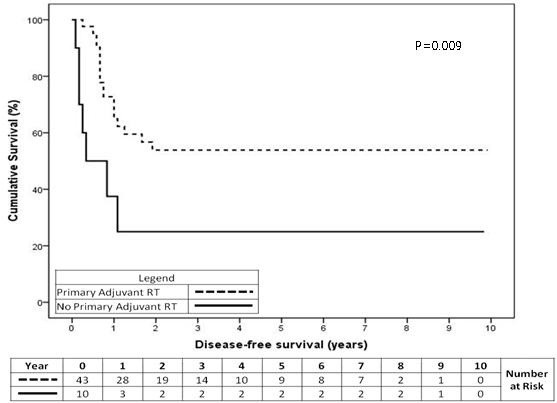
Disease-free survival for the overall cohort of patients having adjuvant RT to their primary site (p=0.009).

Disease-free survival (DFS) was significantly improved for patients having adj RT to their primary site. This result was observed in the overall cohort (Figure 3, p=0.009) and also the subset of patients having stage I and II disease (p=0.048). For the overall cohort, the cumulative 2-year DFS was 54% for the RT group compared with 25% for the no-RT group.

#### Disease-free survival for RT to regional site

**Figure 4:**
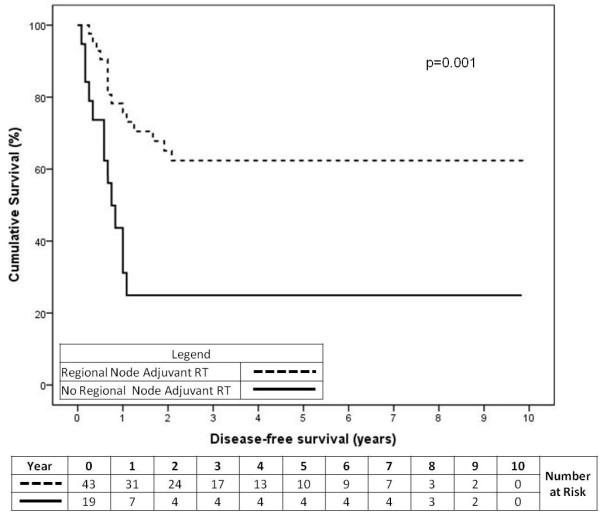
Disease-free survival for the overall cohort of patients having adjuvant or prophylactic RT to their regional nodes (p=0.001).

Similarly, adj RT to the regional nodes was found to significantly improve DFS for the overall cohort (Figure 4, p=0.001). The cumulative 2-year DFS was 64% for the RT group and 25% for the no-RT group.

#### MCC-specific survival

Adj RT to the primary site or to the regional nodes was not found to improve MCCSS in the cohort.

#### Overall survival

Adj RT to the primary site or to the regional nodes was not found to improve OS in the cohort.

### Influence of other factors on survival

The following additional factors were tested for association with DFS, MCCSS, and OS; patient sex, patient age at diagnosis with MCC, patient immune status, primary site of MCC and macroscopic size of the tumour (mm) (Table 3).

**Table 3:** Summary of univariate and multivariate survival results

**Feature**	**Univariate**		**Multivariate**	
	**DFS**	**MCCSS**	**OS**	**OS**
Patient Sex	NS	NS	NS	-
Patient Age (years)	NS	NS	NS	-
Immune Status	NS	p=0.024	p=0.010	HR=2.096, 95%CI: 1.002-4.385, p=0.049*
Primary Site	NS	NS	NS	-
Macroscopic Size (mm)	NS	NS	HR=1.034, 95%CI: 1.006-1.062, p=0.015*	NS
Primary RT	p=0.009	NS	NS	-
Nodal RT	p=0.001	NS	NS	-

*Tested with Cox regression. NS=Not significant.

#### MCC-specific survival and Overall survival

**Figure 5:**
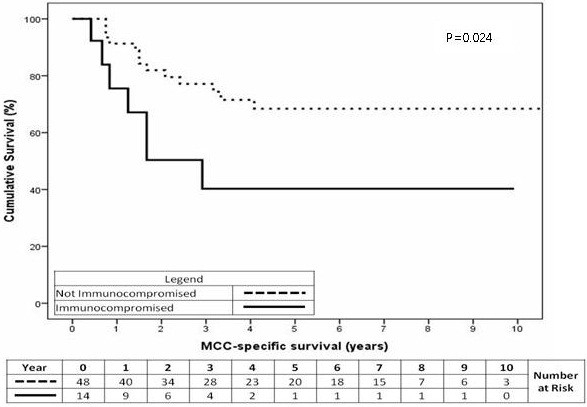
MCC-specific survival for patients according to immune status at initial diagnosis (p=0.024).

**Figure 6:**
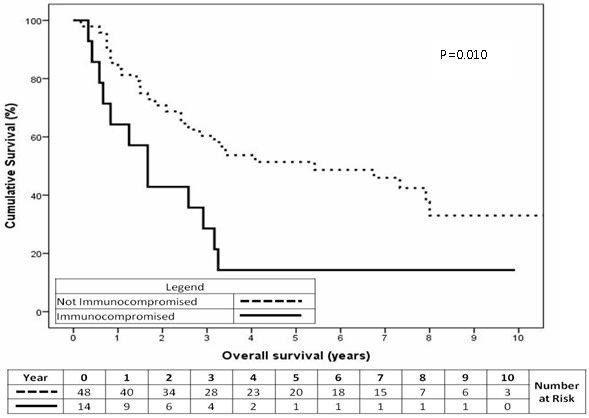
Overall survival for patients according to immune status at the time of initial diagnosis (p=0.010).

Fourteen of 62 patients were immunocompromised at the initial diagnosis. The majority of them had been immunocompromised for more than 10 years. Being immunocompromised at the time of diagnosis was a significant predictor of MCCSS (Figure 5, p=0.024). A patient that was immunocompromised at initial diagnosis had a 2 year cumulative survival of 50% compared with 82% for other patients. Immunocompromised state at the time of diagnosis was a significant predictor of OS (Figure 6, p=0.010). Being immunocompromised at initial diagnosis had a 2 year cumulative OS of 43% compared with 71% for non-immunocompromised patients.

Patient sex, patient age, and primary site were not found to significantly influence MCCSS or OS. The macroscopic size (mm) of the tumour was also a significant predictor of OS on univariate analysis (HR=1.034, 95%CI: 1.006-1.062, p=0.015). However, in a multivariate analysis combining macroscopic size (mm) and immune status, only immune status remained a significant predictor of OS (HR=2.096, 95% CI: 1.002-4.385, p=0.049).

#### Disease-free survival

Analysis of the influence of the site of the primary MCC on DFS was conducted using categories for head & neck, limbs, and trunk (including 1 buttocks case). There was no significant difference in DFS between head & neck and limbs, or between limbs and trunk. Additional factors which were not found to significantly impact DFS included patient sex, patient age, macroscopic size (mm) of the tumour, and whether a patient was immunocompromised at the time of initial diagnosis.

## Discussion

RT as an important part of the treatment paradigm of MCC is supported by our study. Local recurrence free survival and regional recurrence free survival were significantly increased with the addition of RT to the primary site and regional lymph nodes respectively. The addition of these fields was associated with increased DFS for the whole cohort. However, the addition of RT was not found to influence overall or MCCSS. This may be due to small numbers, but may also be because this group of older patients have significant competing risks of death, and have a disease that metastasises early to distant sites out of the treatment volumes of loco-regional RT.

**Figure 7:**
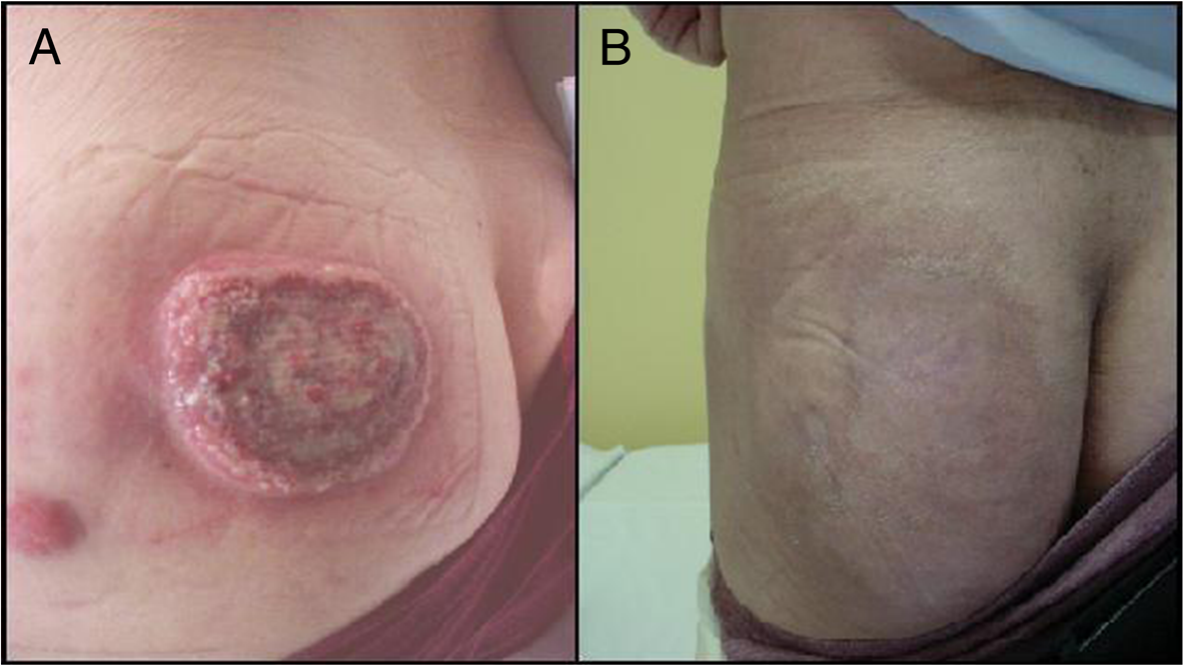
** Merkel Cell Carcinoma radiosensitivity. A**. Ten centimetre deposit of MCC with satellite lesion on buttock. **B**. Same buttock six weeks after thirty Gray of radiotherapy

The relative roles of surgery and RT in MCC are controversial. There are proponents of a predominantly surgical approach while others favour minimal surgery followed by adj RT [3,8,25-27]. Postoperative RT has been strongly recommended by other studies due to its aggressiveness and high risk of recurrence [4,8,28,29] and MCC is known to be highly radiosensitive (Figure 7) [30]. In a review done by Medina-Franco et al. which had 1,024 patients, the mean relapse rate with RT was 10% and 53% without (p=.000001) [5]. The average disease-free period for local recurrence was 7.4 months (range, 4–10 months) [5]. Other studies have found adj RT and adjuvant chemotherapy to be associated with better survival rates [13,31]. Due to the high metastatic potential of MCC, others have suggested systemic therapy and RT rather than radical surgery only [8,15,32]. In patients from the Queensland Radium Institute who were treated with surgery only, all of them had loco-regional relapse and disease-free survival rate at 36 months was 0% [18]. A French trial has recently published that RT to lymph node basin showed a low probability for regional recurrence compared with the observation group who had no regional RT [33]. This study was unfortunately terminated early due to inadequate accrual. 

Immune status at diagnosis was associated with increased MCCSS and OS on multivariate analysis in our study. This is understandable in a disease that is associated with distant spread in the immune-compromised. Immunosuppression appears to be an important risk factor for MCC [1,2,5,6,23]. Immunosuppression enhances the development of MCC in patients with human immunodeficiency virus. They have a relative risk of 13.4 for MCC compared to the general population [22,34]. Studies have shown high risk of developing MCC at a younger age in organ transplantation at presentation [22,35]. Long-term immunosuppressive therapies such as chemotherapy and steroids are associated with aggressive forms of non-melanoma skin cancers [6,23].

Our study does add to the field as it represents a collection of patients treated in a uniform fashion with real conclusions being able to be drawn. However it has significant limitations. It is a retrospective single institutional study, with treatments done in an era where newer advances such as PET and SLNBx have not been incorporated, and so the data could be regarded as obsolete. Polyomavirus was not known in the treatment era of this study. The role of polyomavirus in MCC is one of recent findings that may lead to better therapeutics for MCC [15,36,37]. Our study suggests that local and regional RT are worthwhile treatments for MCC and stratification for immunosuppression should be factored into any future trial design. The limitations of this study include a small sample size and lack of inclusion of new technologies such as PET, SLNBx, and the knowledge of polyomavirus. Randomised studies are needed to guide management and should include RT as treatment and should stratify for immune status at diagnosis.

## Conclusion

This retrospective single institution study of 67 consecutive patients with MCC supports the use of RT to local and regional sites to enhance loco-regional control and hence DFS. RT was not found to increase MCC-specific or overall survival in this disease of older patients that metastasises early to distant sites out of the field of RT. Patients immunocompromised at diagnosis do poorly.

## Competing interests

The authors declare that they have no competing interests.

## Authors’ contributions

SHK and GBF were responsible for initiation of the study. SHK collected the data and drafted the manuscript. LEH performed the statistical analyses. SHK, RYHG and LEH participated in coordination. GBF provided mentorship. SHK and GBF substantially contributed to the editing and revising of the manuscript. All authors have read and approved the final manuscript.
